# Responses to a Social Media Campaign Promoting Safe Fish Consumption Among Women

**DOI:** 10.5888/pcd16.180621

**Published:** 2019-08-01

**Authors:** Jeanette Y. Ziegenfuss, Jennifer Renner, Lisa Harvey, Abigail S. Katz, Kate A. Mason, Patricia McCann, Jeanne Mettner, Katie D. Nelson, Ruth Taswell, Brittney K. Wacholz, Thomas E. Kottke

**Affiliations:** 1 HealthPartners Institute, Bloomington, Minnesota; 2 HealthPartners, Bloomington, Minnesota; 3 Minnesota Department of Health, St. Paul, Minnesota

## Abstract

We used a framework to systematically evaluate which Facebook advertisements promoting safe fish consumption increased traffic to our website. Keeping images and headlines constant, we tested 11 message types in 5 categories between 2 audiences over a 24-hour weekday period. We identified clear preferences in 9 of 10 comparisons and evidence to suggest that more women prefer presentation of question format compared with narratives, marketing compared with patient education copy, and uncertain compared with certain copy. Our test of messages on a social media platform is a quick and inexpensive way to select the most engaging public health messages for broad dissemination.

**Summary**

**What is already known about this topic?**

Social media is a promising tool for disseminating health messages. The framing, content, and context of these messages can affect how well they reach and are used by target populations. Social media offers a cost-effective means of testing messages to ensure selection of those most effective before campaign launch.

**What is added by this report?**

To inform a health education campaign designed to increase awareness about safe fish consumption, we used social media to test 11 different message types within 5 different categories among 2 audiences. In a quick but controlled test, we identified clear preferences in 9 of 10 comparisons and evidence to suggest that more women prefer presentation of question format compared with narratives, marketing compared with patient education copy, and uncertain compared with certain copy. Pregnant women were more likely to prefer a message from experts while nonpregnant women preferred a message from physicians.

**What are the implications for public health practice?**

We demonstrated a quick and effective way to test public health messages. Our findings that some social media messages resonate better than others justify the need for public health practitioners to test messages before campaign launches. To be effective stewards of resources, public health practitioners can use our simple and inexpensive strategy to test messages and identify those with the highest engagement to use in campaigns.

## Objective

Fish contributes to visual and cognitive fetal development (1,2). With aligned missions, the Minnesota Department of Health and HealthPartners, an integrated health system, partnered to develop materials promoting safe fish consumption.

Sixty-eight percent of US adults use Facebook (3–6), which suggests that social media can widely disseminate health messages. Studies measure the reach and engagement of social media messages; however, few have described a methodic evaluation of content before message launch (5,7,8). Because message frame (9), content, and context affect reach (10), messages should be tested in advance. Social media offer a cost-effective means of testing (11). We evaluated the effectiveness of Facebook as a platform for low-resource, rapid message testing about safe fish consumption.

## Methods

We launched a paid Facebook media campaign over a 24-hour weekday period in September 2018 to determine what effect advertising copy had on message success for 2 distinct audiences, pregnant women and nonpregnant women, in our target audience when images and headlines remained constant. The campaign compared responses to 11 message types in 5 categories in our 2 audiences (Table). In each message category, advertisement headlines and images were held constant by audience to ensure that engagement was evaluated solely on the basis of advertisement copy (Figure). An editorial board with combined patient education, marketing, research, evaluation, and content expertise developed messages by using health communication literature, operational interests, and stakeholder opinion as selection criteria. In some instances, identical messages were used for multiple categories, but never within the same category. By using Facebook’s proprietary “Likes and Interests” feature (12), we compared advertisement engagement of women in Minnesota aged 20 to 44 identified by Facebook as having an interest in pregnancy (pregnant, n = 440,000) to a similar group of women without an identified interest in pregnancy (nonpregnant, n = 990,000). The advertisement’s target audience was refined by using this Facebook tool on the basis of user profiles and their connected content. These 2 audiences were chosen because women who are or could become pregnant were the target population for our fish consumption message.

**Table:** Five Message Categories Tested for Engagement (Via Click-Through Rate^a^) Among Pregnant and Nonpregnant Women in Minnesota, 2018

Category	Type	Pregnant Women			Nonpregnant Women		
		Message	Click-through Rate, %	*P* Value^b^	Message	Click-through Rate, %	*P* Value^b^
Narrative technique	Narrative	Omega-3 fatty acids in fish are a building block for a baby’s brain and eyes.	0.21	<.001	Learn how eating fish may benefit your health.	0.17	<.001
	Question format	How can omega-3 fatty acids in fish affect a baby’s brain and eyes?^c^	0.39		How can eating fish benefit your health?^c^	0.29	
Discipline of approach	Patient education	Omega-3 fatty acids in fish are a building block for a baby’s brain and eyes.	0.21	<.001	Learn how eating fish may benefit your health.	0.17	<.001
	Marketing	Omega-3 fatty acids in fish — a building block for a baby’s brain and eyes!^c^	0.30		Eating fish benefits your health — learn how!^c^	0.29	
Certainty	Certain	Omega-3 fatty acids in fish are a building block for a baby’s brain and eyes.	0.21	.006	Learn how eating fish will benefit your health.	0.17	<.001
	Uncertain	Omega-3 fatty acids in fish can be a building block for a baby’s brain and eyes.^c^	0.28		Learn how eating fish may benefit your health.^c^	0.22	
Framing	Gain	Omega-3 fatty acids in fish help with a baby’s brain and eye development.	0.31	.30	Learn how eating fish could benefit your health.^c^	0.26	<.001
	Risk	Avoiding fish while you are pregnant may negatively impact your baby’s brain and eye development.	0.34		Avoiding fish may mean you are missing out on important nutrients that are hard to get elsewhere.	0.10	
Source	Named clinician	“Omega-3 fatty acids in fish are a building block for a baby’s brain and eyes.” — Dr. Jane Smith, OB/GYN	0.30	<.001	“Eating fish may benefit your health.” —Dr. Jane Smith, OB/GYN	0.24	<.001
	Physicians	Physicians say that Omega-3 fatty acids in fish are a building block for a baby’s brain and eyes.	0.25		Physicians say that eating fish may benefit your health.^c^	0.37	
	Experts	Experts say that Omega-3 fatty acids in fish are a building block for a baby’s brain and eyes.^c^	0.40		Experts say that eating fish may benefit your health.	0.30	

Abbreviation: OB/GYN, obstetrician/gynecologist.

a Calculated by dividing the number of people who clicked on the advertisement by the number of people who saw it.

b *P* value calculated by χ^2^ test comparing each message type’s click-through rate in audience.

c Messages in each message type that audiences found most engaging.

**Figure:**
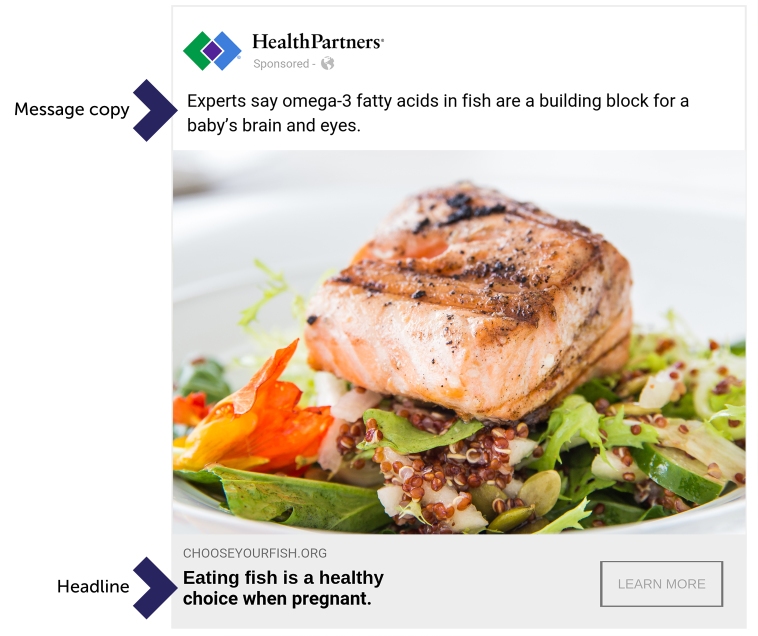
Example Facebook advertisement used in the message testing campaign. The “expert” source ad shown here was most engaging for the audience of pregnant women.

Advertisements were released simultaneously by using Facebook’s daily reach feature to minimize the chance that an advertisement was seen twice. Click-through rate (CTR), calculated by dividing the number of people who clicked on the advertisement by the number of people who saw it, measured engagement. For each audience, the CTR of each message type was compared by using χ^2^ tests. Significance was set at α = 0.05. Personnel and direct Facebook costs were summed to determine total implementation cost.

## Results

The Facebook campaign reached 76,592 pregnant women and 86,816 nonpregnant women. No other demographic information was collected. We successfully identified a preference in 9 of 10 total tests (*P* < .05). The copy that resonated most with pregnant women had an expert source, with a 0.40% CTR (Table). The least engaging advertisements (CTR of 0.21% each) for this group were of the “Narrative,” “Certain,” and “Patient Education” message type: “Omega-3 fatty acids in fish are a building block for a baby’s brain and eyes.” The only set of advertisements that did not show clear preference were gain (ie, promoting the benefits of fish) and risk (ie, warning about negative effects of not eating enough fish) frame. For nonpregnant women, the advertisement with a physician source was most engaging with a 0.37% CTR, whereas the advertisement with risk framing was least engaging (CTR = 0.10%). For both audiences, question format, marketing, and uncertain advertisements were more engaging than their foils. Direct costs to run the advertisements on Facebook combined with 13 personnel hours needed to implement the tests and summarize the results brought the total implementation costs to under $2,500. This did not include image costs, because HealthPartners has an organizational subscription to the source of advertisement images used, or time to develop the advertisement copy.

## Discussion

A team of multidisciplinary experts generated sets of test messages derivative of 1 common message across 5 categories, for a total of 11 message types. In a quick but controlled test, we identified clear preferences in 9 of 10 comparisons and evidence to suggest that more women prefer presentation of question format compared with narratives, marketing compared with patient education copy, and uncertain compared with certain copy. Pregnant women were more likely to prefer a message from experts and nonpregnant women preferred a message from physicians.

Future social media campaigns for safe fish consumption in Minnesota will use the messages and strategies found through our testing to be most engaging for each of our target populations. Although the findings about which message strategies are most useful for our topic and setting are limited to women who use Facebook and reside in Minnesota, the strategy to find relevant messages can be applied to any topic and setting. We recognize that there is no limit to the message strategies that can be tested, but we chose those that were directly pursuant to the literature, operational considerations, or stakeholder opinion. Furthermore, we do not offer an explanatory model for why the identified strategies were most effective or why the results differed by subpopulation. Nonetheless, our test of messages on a social media platform was a quick and inexpensive way to select the most engaging public health messages for broad dissemination.
